# Sex-specific preservation of neuromuscular function and metabolism following systemic transplantation of multipotent adult stem cells in a murine model of progeria

**DOI:** 10.1007/s11357-023-00892-5

**Published:** 2023-08-03

**Authors:** Seth D. Thompson, Kelsey L. Barrett, Chelsea L. Rugel, Robin Redmond, Alexia Rudofski, Jacob Kurian, Jodi L. Curtin, Sudarshan Dayanidhi, Mitra Lavasani

**Affiliations:** 1 Shirley Ryan AbilityLab, 355 E. Erie St, Chicago, IL 60611 USA; 2 Department of Physical Medicine and Rehabilitation, Northwestern University, Chicago, IL 60611 USA; 3 Northwestern University Interdepartmental Neuroscience (NUIN) Graduate Program, Northwestern University, Chicago, IL 60611 USA; 4 Department of Biomedical Engineering, Northwestern University, Chicago, IL 60611 USA

**Keywords:** Progeria, Aging, Regeneration, Cell therapy, Sex differences, Metabolism, Skeletal muscle, Muscle fatigue, Neuromuscular tissues

## Abstract

Onset and rates of sarcopenia, a disease characterized by a loss of muscle mass and function with age, vary greatly between sexes. Currently, no clinical interventions successfully arrest age-related muscle impairments since the decline is frequently multifactorial. Previously, we found that systemic transplantation of our unique adult multipotent muscle-derived stem/progenitor cells (MDSPCs) isolated from young mice—but not old—extends the health-span in DNA damage mouse models of progeria, a disease of accelerated aging. Additionally, induced neovascularization in the muscles and brain—where no transplanted cells were detected—strongly suggests a systemic therapeutic mechanism, possibly activated through circulating secreted factors. Herein, we used ZMPSTE24-deficient mice, a lamin A defect progeria model, to investigate the ability of young MDSPCs to preserve neuromuscular tissue structure and function. We show that progeroid ZMPST24-deficient mice faithfully exhibit sarcopenia and age-related metabolic dysfunction. However, systemic transplantation of young MDSPCs into ZMPSTE24-deficient progeroid mice sustained healthy function and histopathology of muscular tissues throughout their 6-month life span in a sex-specific manner. Indeed, female—but not male—mice systemically transplanted with young MDSPCs demonstrated significant preservation of muscle endurance, muscle fiber size, mitochondrial respirometry, and neuromuscular junction morphometrics. These novel findings strongly suggest that young MDSPCs modulate the systemic environment of aged animals by secreted rejuvenating factors to maintain a healthy homeostasis in a sex-specific manner and that the female muscle microenvironment remains responsive to exogenous regenerative cues in older age. This work highlights the age- and sex-related differences in neuromuscular tissue degeneration and the future prospect of preserving health in older adults with systemic regenerative treatments.

**Supplementary Information:**

The online version contains supplementary material available at 10.1007/s11357-023-00892-5.

## Introduction

Sarcopenia is a significant chronic clinical condition prevalent in the aging population that progressively reduces muscle function. Hallmarks include both cellular impairments, such as reduced muscle fiber size, motoneuron innervation, and mitochondrial function, and functional impairments, such as accelerated loss of muscle strength and mobility [1–3]. This contributes to lower extremity muscle fatigue and impaired balance, resulting in a dramatically greater risk of falls and injuries [4]. Indeed, unintentional injury, mostly due to falls, is the seventh leading cause of death in those 65 years of age or older [5]. Sarcopenia affects more than 45% of Americans over the age of 60, including 31% of females and 64% of males, with annual healthcare costs over $18 billion [6]. However, sex differences associated with multifactorial age-related muscle decline are still not well understood. Selection bias of male mice in a large range of biomedical studies has likely contributed to this gap in knowledge [7]. To date, sex has been identified as a confounding factor in many age-related diseases and attempted therapies, particularly in relation to muscle wasting [8–11]. Consequently, there are currently no effective clinical interventions that halt or reverse these potentially life-threatening changes in either sex.

At a cellular level, the sarcopenic muscle presents a smaller myofiber cross-sectional area [12] without a reduction in myofiber number [13]. The reduced size of aged muscle fibers is also associated with a decline in mitochondrial function and abundance [3, 14]. Extensive investigation on the role of resident monopotent muscle progenitor cells (i.e., satellite cells) in aging mammalian skeletal muscle has demonstrated an age-associated decline in satellite cell number that may directly contribute to a smaller muscle fiber size [15, 16]. In addition, satellite cells in vivo frequently experience a shift toward fibrogenic activity during aging, leading to tissue remodeling and increased fibrosis, which can impair function [17]. However, satellite cell ablation in mice at a young age has not been shown to accelerate the onset or increase severity of sarcopenia [18]. This suggests that other, likely extrinsic, factors are responsible for driving sarcopenia. Therefore, this evidence suggests a need to investigate the therapeutic potential of other biological interventions (e.g., stem cells or growth factors) as alternatives for reversing sarcopenia.

To date, local stem cell transplantation as a common strategy has shown insufficient improvement in muscle structure and function due to limited cell migration and impact beyond the proximity of the injection site [19–26]. Importantly, due to the multifactorial nature of aging (e.g., genetic, environmental, lifestyle), targeting individual muscles/tissues to regain function is now increasingly being recognized as a fruitless endeavor [27]. Instead, strategies that combat aging systemically have begun to receive more attention. This has been elegantly reflected through heterochronic parabiosis of mice, revealing that circulating factors from young mice improve function and regeneration of many tissues, including muscle, in old mice [28, 29]. Similar results were observed with engrafting aged muscle into young hosts, providing the aged muscle with a young niche [28, 30–33]. However, the underlying mechanisms remain elusive and neither of these methods provides a clinically translatable approach.

Muscle-derived stem/progenitor cells (MDSPCs) are adult multipotent stem cells isolated from skeletal muscle via a modified preplate technique [34]. MDSPCs isolated from young mice have the capacity for long-term proliferation, self-renewal, multi-lineage differentiation into myogenic, neurogenic, glial, osteogenic, adipogenic, and chondrogenic lineages, are resistant to oxidative stress, and can induce neovascularization—all of which likely contribute to their ability to promote regeneration [23, 35–39]. Local MDSPC transplantation has been used as a candidate cell therapy for regeneration after musculoskeletal injuries in young mice [23–26]. This is primarily due to their inherent plastic ability to alter phenotypic commitment in response to environmental cues, which can support host cell recruitment, and/or the growth factors they release that promote regeneration [23, 26, 40, 41]. Furthermore, our previous study demonstrated that systemic transplantation of young—but not old—MDSPCs into animal models of progeria, a disease of accelerated aging, doubles their life-span, extends their health-span, and improves muscle composition [42]. More recently, we have shown this restorative therapy is capable of preventing and reversing osteoarthritis, reducing pro-inflammatory senescence-associated secretory phenotype (SASP) factors, and improving functional gait metrics in progeroid and naturally aged mice [43, 44]. Although the mechanisms involved are still unknown and under investigation, neovascularization and immune modulation—in tissues where no transplanted cells were detected—strongly suggest tissue rejuvenation is mediated through systemically circulating paracrine and endocrine factors secreted by MDSPCs.

**Fig. 1:**
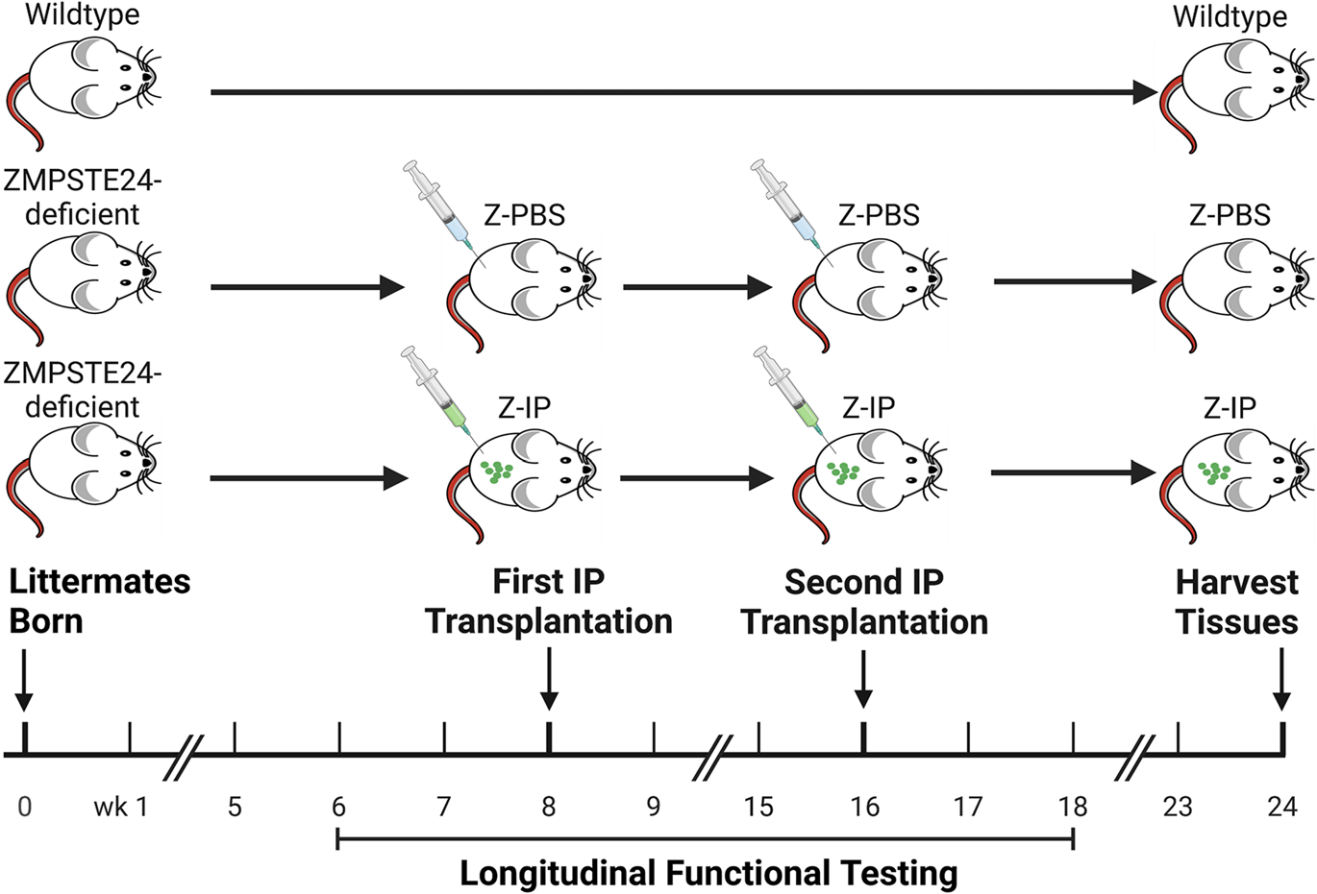
Schematic illustrating the in vivo experimental design. Littermate male and littermate female wildtype (WT) and progeroid ZMPSTE24-deficient mice were used in all three groups. Functional testing was initiated prior to treatment for baseline measurements starting at 6 weeks (wks) of age and after treatment up to 18 wks of age. Sex-matched ZMPSTE24-deficient mice littermates were intraperitoneally (IP) injected with either PBS (Z-PBS) or young MDSPCs (Z-IP) at 8 wks of age and again at 16 wks of age. Tissues were harvested at approximately 6 months (24 wks) of age. Schematic was created with BioRender.com

ZMPSTE24-deficient mice are a model of Hutchinson-Gilford progeria syndrome (HGPS), which recapitulates many hallmarks of normal human aging [45, 46] including weight loss, sarcopenia, impaired muscle contraction, kyphosis, grip abnormality, cardiomyopathy, peripheral neuropathy, osteoarthritis, and reduced neuromuscular performance [43, 47–50]. The short life span of the ZMPSTE24-deficient progeroid mice (6 months) provides a unique opportunity to test systemic preventative interventions against aging-related diseases and obtain longitudinal life span data. Herein, we examined the extent of neuromuscular degeneration in ZMPSTE24-deficient mice and whether healthy muscle tissue structure and function can be preserved throughout their life span by systemic transplantation of young MDSPCs prior to manifestation of severe muscle degeneration (Fig. 1). Our findings indicate that ZMPSTE24-deficient mice exhibit increased muscle fatigability and that multiple muscle groups show significant reduction in muscle fiber size, mitochondrial function, fatigue-resistant fibers, and innervation. Importantly, systemic transplantation of young MDSPCs preserved muscle function and composition in a sex-specific manner; this includes reducing muscle fatigue and fibrosis while increasing muscle fiber size, fatigue-resistant fibers, and innervation, in female mice but not in male mice. These novel findings provide strong evidence that systemic transplantation of young multipotent MDSPCs has a therapeutic effect on aging muscle function and histopathology and that sex is a major modulator of their success to preserve healthy muscle.

## Materials and methods

### MDSPC isolation

Young WT MDSPCs were isolated from the hindlimb skeletal muscle of 21-day-old female mice via a modified preplate technique, using previously published methods [34]. MDSPCs were cultured and expanded for transplantation in proliferation medium (PM): high glucose DMEM supplemented with 10% horse serum, 10% fetal bovine serum (FBS), 1% penicillin–streptomycin (all from Invitrogen), and 0.5% chick embryo extract (CEE, Accurate Chemical). Cells are cultured on collagen type I-coated flasks.

### Animal husbandry

All animal experiments were performed with the approval of the Northwestern University Institutional Animal Care and Use Committee. Seventeen-day-old mice were ear tagged, and tail snips were collected and mailed through the Northwestern University in-house system to Transnetyx for genotyping. Primers and PCR conditions for genotyping *Zmpste24*^−/−^ mice have been previously published [50]. Mice were maintained in a pathogen-free facility at 23–24 °C under a 12-h light, 12-h dark regimen and fed ad libitum a standard chow which is gamma irradiated. Mice were always studied in sibling pairs to minimize environmental variables. Only if breeding produced two or more *Zmpste24*^−/−^ mice would the litter be used in the study so that one mutant animal is provided the experimental treatment, and the other receives vehicle only. ZMPSTE24-deficient mice were generated through breeding of heterozygous pairs, kindly provided by Dr. Carlos-Lopez Otin.

### Cell transplantation

MDSPCs suspended in 50 µL of buffered saline (PBS) were transplanted IP into 2-month-old *Zmpste24*^−/−^ mice at 2 × 10^5^ cells per gram body weight. For each MDSPC transplanted animal, a sex-matched littermate mutant animal was injected with vehicle only (PBS). Both male and female mice were included in the transplantation study. The injections were repeated 2 months later at 4 months of age. Mice were sacrificed at the end of their natural life span, approximately 5–6 months of age, and tissues were collected.

### Functional assessment

Muscle fatigue was measured using the four-limb hang test [55]. Mice were placed on a rectangular grid of ½″ × ½″ galvanized 19GA hardware cloth and then inverted approximately 1 ft over a floor cushioned with enviro-dri paper topped with a surgical drape in an enclosed rectangular plastic container. The amount of time the mice were able to hang was recorded, up to a maximum of 5 min. Each mouse was measured three times per time point with no less than 15 min in between each test. To calculate the hanging impulse, the mouse’s average hang time(s) is multiplied by their weight in grams and divided by 100.

### Muscle fiber analysis

The muscle fiber cross-sectional area was assessed in ZMPSTE24-deficient mice by immunohistochemically (IHC) labeling GS muscle sections for dystrophin, the anchor protein that attaches the myofiber cytoskeleton to the plasma membrane (sarcolemma), using previously established protocols [23, 42, 74]. The cross-sectional area was quantified from images taken with a Leica DM6000 automated upright microscope using a × 20 plan APO objective and analyzed using identical program thresholding parameters for fiber selection and area measurements in NIS-Elements computational software to avoid measurement bias.

Muscle fiber type distribution was assayed as previously described [66]. Briefly, cross sections of GS and soleus muscles were IHC labeled with an antibody cocktail for myosin heavy chain (MHC) type 1, type 2A, and type 2B, with the vacant unstained fibers being determined to be type 2X (this was tested and verified on one muscle from each group as recommended by the referenced protocol). Fiber type distributions of medial-GS and soleus muscles were quantified from images taken with a Leica DM6000 automated upright microscope using a × 20 or × 40 plan APO objective and counted manually.

### Muscle fibrosis

Muscle fibrosis was assessed by double-staining GS muscle sections with sirius red and fast green. Fibrosis was quantified from light microscopy images taken with a Leica DM6000 automated upright microscope using a × 20 plan APO objective compiled into a total muscle section mosaic in Adobe Photoshop, and NIS-Elements software was used to determine the total muscle area and percentage of muscle area composed of collagen using identical threshold parameters across all samples.

### Muscle innervation

AChR number and morphology (i.e., length and area) were quantified following staining of GS muscles with a fluorescently conjugated α-BTX and co-labeled with anti-dystrophin antibodies. Muscle sections were imaged with a Leica DM6000 automated upright microscope using a × 40 plan APO objective and analyzed using identical program thresholding parameters for AChR selection and area measurements in NIS-Elements computational software.

### Mitochondrial respirometry

High-resolution respirometry was performed using an Oroboros O2K (Oroboros Instruments, Innsbruck, Austria) per established protocols [75–77]. Briefly, quadriceps muscle was harvested immediately after euthanasia and placed in preservation solution (BIOPS; 2.77 mM CaK2EGTA, 7.23 mM K2EGTA, 5.7 mM Na2ATP, 6.56 mM MgCl2, 20 mM taurine, 15 mM Na2Phosphocreatine, 20 mM imidazole, 0.5 mM DTT, and 50 mM MES). The quadriceps muscles were then mechanically separated in BIOPS on ice under a dissecting microscope to obtain replicates of around 2–3 mg and permeabilized with 50 μg/mL saponin for 30 min followed by a 10 min wash in mitochondrial respiration media [MiR05; 0.5 mM EGTA, 3 mM MgCl2, 60 mM K-lactobionate, 20 mM taurine, 10 mM KH2PO4, 20 mM 4-(2-hydroxyethyl)-1-piperazineethanesulfonic acid, 110 mM sucrose, and 1 g/L fatty acid-free bovine serum albumin]. All data were collected at 37 °C in hyperoxygenated (200–400 mM) conditions in MiR05 to avoid limitations with oxygen diffusion. The sequential substrate-uncoupler-inhibitor titration (SUIT) respiration protocol used to test for maximal phosphorylation and electron transport chain capacity of complex-I– and complex-II–mediated respiration was 5 mM pyruvate, 0.5 mM malate, 5 mM adenosine diphosphate (ADP), 10 mM glutamate, 10 mM cytochrome c, 10 mM succinate, followed by 0.5 mM titration carbonyl cyanide m-chloro phenyl hydrazine (CCCP), 0.5 mM rotenone, and 2.5 mM antimycin A (Ama). Any replicates that were higher than 10% following the addition of cytochrome c (cytochrome c test) were considered to be over permeabilized, and the data were not used. The state of respiration without ADP was leak, after addition of ADP was state 3 respiration, after glutamate was complex-I (+ III + IV + V) mediated phosphorylation capacity (Phos CI), that after succinate was complex-I + II (+ III + IV + V), i.e., maximal phosphorylation capacity (Phos CI + II), and the state after addition of CCCP (uncoupler) was maximal electron transport chain capacity (ETC CI + II). Rotenone and antimycin A were used as inhibitors for complex-I and complex-III, respectively, to assess functionality of permeabilized muscle fiber mitochondria.

### Citrate synthase rate assay

Following a previously published method [78, 79], flash-frozen quadriceps muscles were powdered using liquid nitrogen–chilled mortar and pestle and maintained under cold conditions using dry ice. Powdered tissue was homogenized in Zheng buffer (210 mmol/L mannitol, 70 mmol/L sucrose, 5 mmol/L 4-[2-hydroxyethyl]-1-piperazineethanesulfonic acid [HEPES], and 1 mmol/L ethylene glycol-bis(β-aminoethyl ether)-N,N,N′,N′-tetraacetic acid (EGTA) (pH 7.2)) (ratio 1:10). Tissue was homogenized using clean glass-on-glass conical tissue grinders, kept on ice with 16 slow, controlled up-down strokes at 500 rpm followed by centrifugation at 600 g at 4 °C for 10 min. Protein concentration of the supernatant was determined using a Pierce™ bicinchoninic acid protein assay kit per manufacturer’s instructions (Thermo Fisher Scientific, Waltham, MA, USA). Aliquots were created for citrate synthase assay using similar protein concentrations of 1.0 μg/μL across the samples. Tris (tris[hydroxymethyl]aminomethane) buffer (pH 8.0) was used with 12.5 mmol/L acetyl coenzyme A as the substrate for the tricarboxylic acid cycle with 5 mmol/L oxaloacetic acid and 1 mmol/L 5,5′-dithiobis 2-nitrobenzoic acid (DTNB) as the electron acceptors. Rate of increase in absorbance over 3 min due to reduction of DTNB after the addition of oxaloacetic acid was used to determine the activity rate. Assays were conducted using 10 μg of protein/well at a wavelength of 412 nm with an extinction coefficient of 13.6 mmol/L/cm.

### Statistics

Statistical analyses were carried out using the SigmaPlot (Jandel Scientific v14.0) and GraphPad Prism (v9) software packages. Two-tailed or one-tailed Student’s *t*-test was used for comparisons between WT and saline-transplanted cohorts and saline- and cell-transplanted cohorts, respectively. Bar graphs, line graphs, and frequency distributions are expressed as the mean ± SEM, and *p* ≤ 0.05 was considered significant.

## Results

### ZMPSTE24-deficient mice exhibit increased muscle fatigability, smaller muscle fiber size, and reduced innervation

**Fig. 2:**
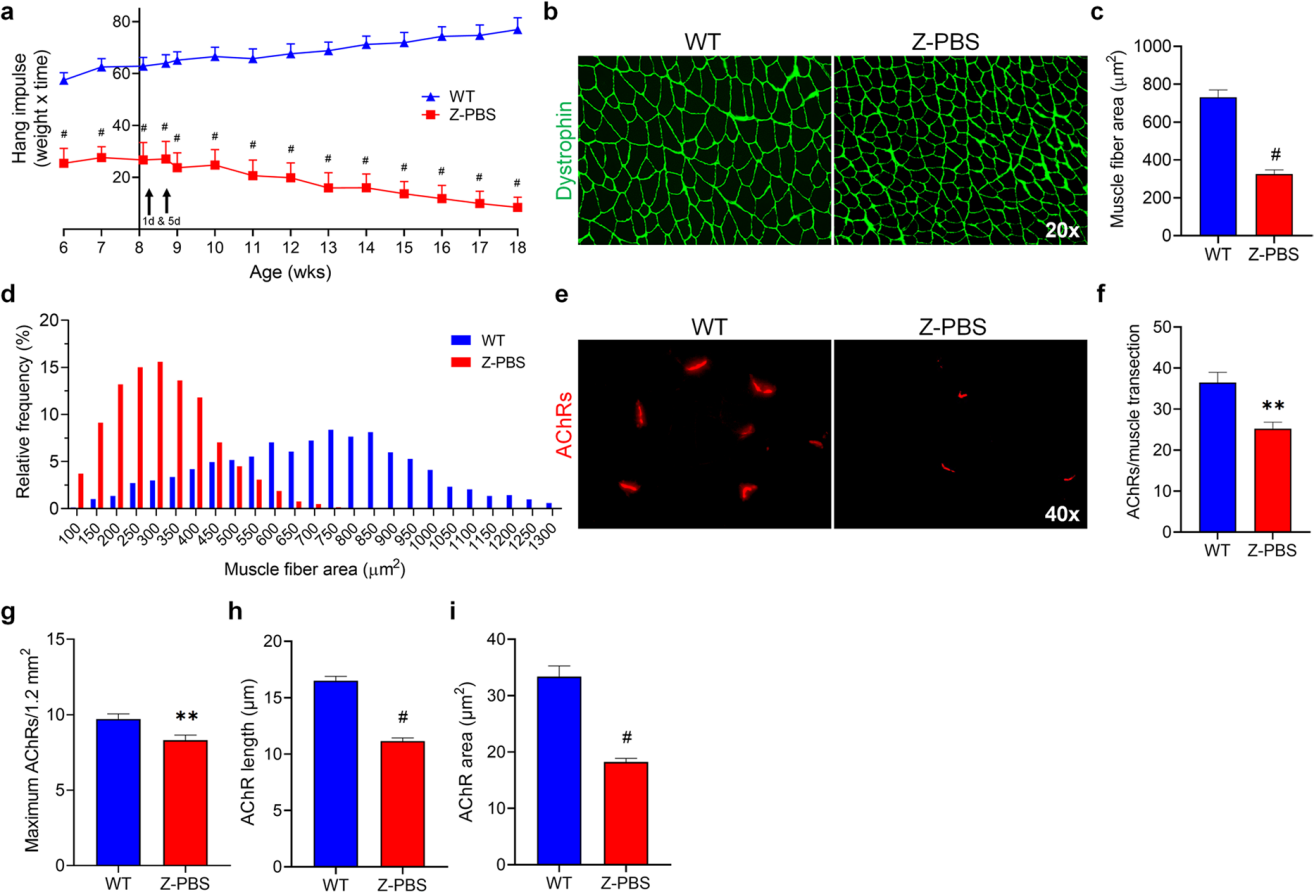
Progeroid ZMPST24-deficient mice have increased muscle fatigability, reduced muscle fiber area, and decreased muscle innervation. **a** Hanging impulse (weight × hang time) of wildtype (WT) control mice compared to age-matched ZMPSTE24-deficient littermates intraperitoneally transplanted with PBS (Z-PBS) measured by the four-limb hang test. Y-intercept is at 8 weeks (wks), the time of PBS transplantation, with time points at 1-day (d) and 5-d post-transplantation. **b** Representative images of dystrophin (green) in gastrocnemius muscles from WT and Z-PBS mice. **c** Quantification of muscle fiber area in WT and Z-PBS mice. **d** Histogram of muscle fiber cross-sectional area frequencies in WT and Z-PBS mice. **e** Representative images of α-bungarotoxin (α-BTX) labeled acetylcholine receptors (AChRs, red) in gastrocnemius muscles from WT and Z-PBS mice. **f** Graphs indicating the number of total AChRs per muscle cross section and **g** the average density of AChRs from WT and Z-PBS gastrocnemius muscles. **h** Quantification of average AChR length and **i** AChR area in WT and Z-PBS mice. Error bars indicate ± SEM, *n* = 8 for WT and *n* = 8 to 11 for Z-PBS. **p ≤ 0.01 and ^#^*p* ≤ 0.001 using two-tailed, unpaired Student’s *t*-test or Welch’s unequal variance *t*-test

To substantiate that progeroid ZMPSTE24-deficient mice present with functional pathologies of sarcopenia, a longitudinal study of muscle fatigue was performed using the four-limb hang test (total and sex-specific animal numbers for all experiments are listed in Supplementary Information Table S1). Quantification of hanging impulse (mouse weight × hang time) demonstrated a significant increase of muscle fatigability in negative control ZMPSTE24-deficient mice intraperitoneally (IP) transplanted with PBS (Z-PBS) compared to age-matched wildtype littermates (WT; Fig. 2a, ^#^*p* ≤ 0.001). The difference in muscle fatigability was evident from the initial time point at 6 weeks (wks) of age and continued throughout their life span. At the end of the Z-PBS 6-month life span, gastrocnemius (GS) muscles were collected from Z-PBS and WT mice and muscle cross sections were labeled for dystrophin (green; Fig. 2b). Compared to WT littermates (Fig. 2c; 731.0 µm^2^), the average Z-PBS muscle fiber area was significantly smaller (325.2 µm^2^; ^#^*p* ≤ 0.001). A frequency histogram of muscle fiber cross-sectional areas clearly illustrates the extensive shift toward smaller fiber areas in muscles from Z-PBS mice in comparison to WT mice (Fig. 2d). GS muscles were also labeled with α-bungarotoxin (α-BTX, red) to identify acetylcholine receptors (AChRs) as a marker to evaluate muscle innervation (Fig. 2e, Fig. S1). The average number of Z-PBS AChRs per entire muscle cross section (Fig. 2f; 25.2; **p ≤ 0.01) and density (Fig. 2g; 8.3; **p ≤ 0.01) were significantly reduced in comparison to age-matched WT mice (36.5 and 9.7, respectively). Morphometric analysis was performed on AChRs, illustrating Z-PBS have not only a reduction in AChR quantity but also quality, as measured by AChR length (Fig. 2h; 11.1 µm vs WT 16.5 µm; ^#^*p* ≤ 0.001) and area (Fig. 2i; 18.2 µm^2^ vs WT 33.4 µm^2^; ^#^*p* ≤ 0.001). Together, these results demonstrate the age-related neuromuscular tissue deficiencies in progeroid ZMPSTE24-deficient mice are comparable to those of sarcopenia.

### ZMPSTE24-deficient skeletal muscles have reduced mitochondrial function and fatigue-resistant fiber types

**Fig. 3:**
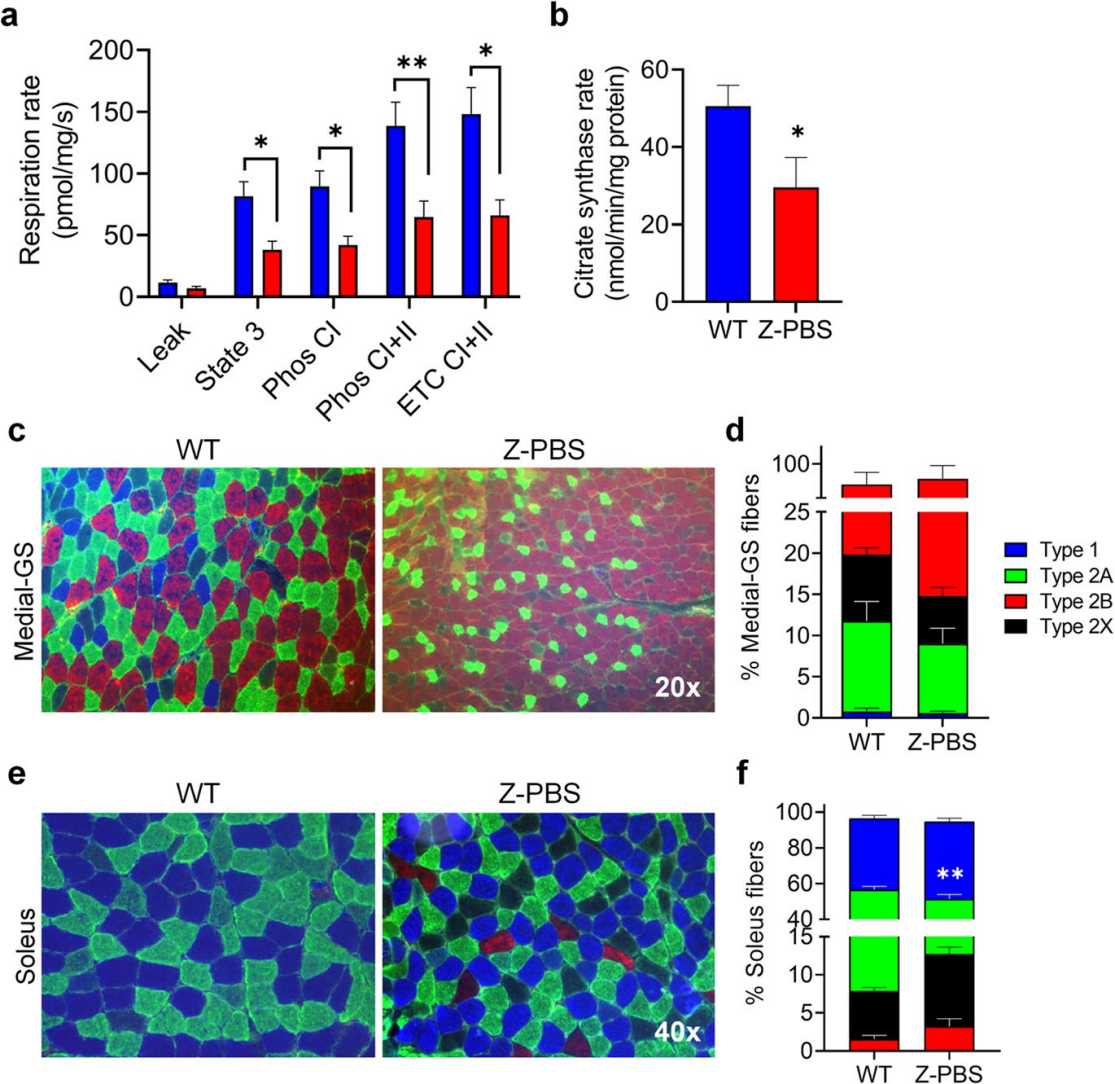
Mitochondrial dysfunction and loss of fatigue-resistant muscle fiber types in ZMPSTE24-deficient mice. **a** Quantification of mitochondrial respiration rate measured from quadriceps muscles of WT and age-matched Z-PBS littermate mice. **b** Graph indicating citrate synthase rate measured from quadriceps muscles of WT and Z-PBS mice. **c** Representative images of fiber type labeling in medial gastrocnemius (medial-GS) muscles from WT and Z-PBS mice. **d** Quantification of medial-GS muscles from WT and Z-PBS mice immunohistochemically labeled for type 1 (slow fatigue-resistant; blue), type 2A (fast fatigue-resistant; green), type 2X (fast fatigable; black), or type 2B (fast highly fatigable; red). **e** Representative images of fiber type labeling in soleus muscles from WT and Z-PBS mice. **f** Quantification of immunohistochemical fiber type analysis from WT and Z-PBS soleus muscles. Error bars indicate ± SEM, *n* = 8 for WT in all graphs and *n* = 5 (**a** and **b**) and *n* = 8 (**d** and **f**) for Z-PBS. *p ≤ 0.05 and **p ≤ 0.01 using two-tailed, unpaired Student’s *t*-test or Welch’s unequal variance *t*-test

Since diminished mitochondria function is also a hallmark of sarcopenia [3, 14], mitochondrial respirometry and citrate synthase levels were measured from quadriceps muscles of WT and Z-PBS mice. Respiration rates were significantly lower in state 3, Phos CI, Phos CI + II, and ETC CI + II of Z-PBS mice (Fig. 3a, *p ≤ 0.05, **p ≤ 0.01). Citrate synthase activity was also significantly decreased in Z-PBS mice (Fig. 3b; 29.5 nmol/min/mg protein) compared to WT (50.5 nmol/min/mg protein; *p ≤ 0.05). Since mitochondrial function is markedly related to muscle fiber types [51], histological fiber type analysis was performed on the medial gastrocnemius (medial-GS; Fig. 3c) and soleus muscles (Fig. 3e), due to their mixed fiber content. Muscle fibers were immunohistochemically labeled as type 1 (slow fatigue-resistant; blue), type 2A (fast fatigue-resistant; green), type 2X (fast fatigable; black), or type 2B (fast highly fatigable; red). The medial-GS of Z-PBS mice exhibited more anaerobic/fatigable 2B fibers, which are the least dense in mitochondria, compared to WT, but this was not statistically significant (Fig. 3d; 81.7% and 75.3%, respectively; *p = 0.151). However, in the soleus muscle, Z-PBS had significantly fewer 2A fibers (Fig. 3f; 38.7%; **p ≤ 0.01)—which are denser in mitochondria content than other type 2 fibers—compared to WT (48.6%), as well as fewer combined fatigue-resistant fibers (type 1 + type 2A; 82.0% vs WT 88.6%; **p ≤ 0.01). These data demonstrate significant age-related metabolic dysfunction in multiple skeletal muscles of progeroid ZMPSTE24-deficient mice compared to their WT littermates.

### Systemic transplantation of young MDSPCs reduces muscle fatigue in female ZMPSTE24-deficient mice

**Fig. 4:**
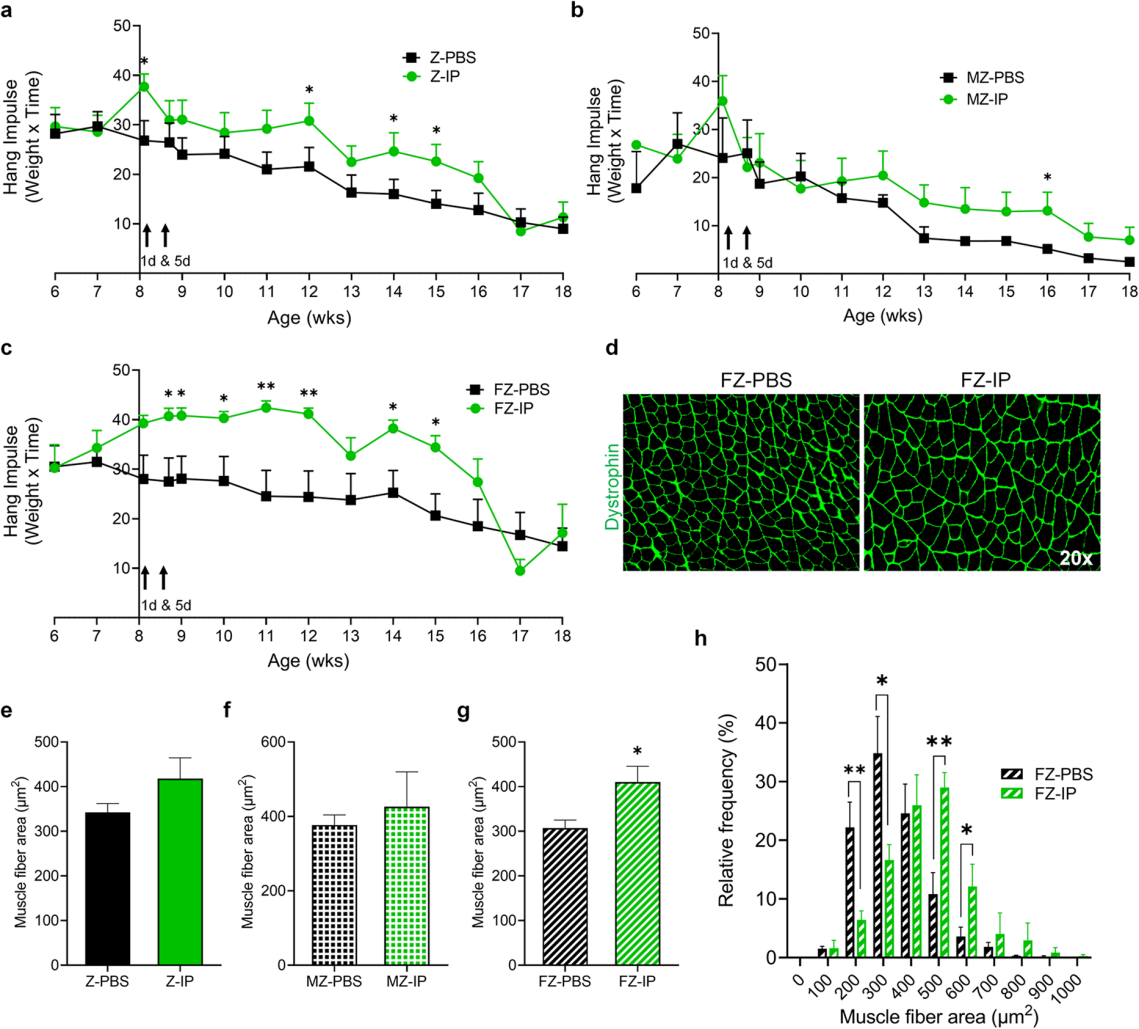
Systemic transplantation of young MDSPCs reduces muscle fatigability and increases muscle fiber area in female ZMPSTE24-deficient mice. **a** Analysis of hanging impulse (weight × hang time) measurements from ZMPSTE24-deficient mice intraperitoneally transplanted with either PBS (Z-PBS) or young MDSPCs (Z-IP) measured by the four-limb hang test. Y-intercept is at 8 weeks (wks), the time of young MDSPC and PBS transplantation, with time points at 1-day (d) and 5-d post-transplantation. **b** Graphs indicating hanging impulse in male Z-PBS (MZ-PBS) and Z-IP (MZ-IP) mice and **c** female Z-IP (FZ-IP) and Z-PBS (FZ-PBS) mice. **d** Representative images of dystrophin (green) in gastrocnemius muscles of Z-PBS and Z-IP mice. **e** Quantification of gastrocnemius muscle fiber area in Z-PBS and Z-IP mice, **f** MZ-PBS and MZ-IP mice, and **g** FZ-PBS and FZ-IP mice. **h** Graph indicating the muscle fiber cross-sectional areas frequency distributions in FZ-PBS and FZ-IP mice. Error bars indicate ± SEM, *n* = 8 for Z-PBS, *n* = 7 to 8 for Z-IP, *n* = 4 for MZ-PBS and MZ-IP, and *n* = 3 to 4 for FZ-PBS and FZ-IP. *p ≤ 0.05 and **p ≤ 0.01 using one-tailed, unpaired Student’s *t*-test or Welch’s unequal variance *t*-test

To determine whether systemic transplantation of young MDSPCs prevents age-associated muscle fatigability in ZMPSTE24-deficient mice, we performed a longitudinal hang test study initiated at approximately 6 wks of age—2 wks prior to the first IP transplantation with either young MDSPCs (Z-IP) or PBS (Z-PBS) (Fig. 1). Measurements were taken on a weekly basis, except for the week of injection, which included additional tests at 1- and 5-d post-injection. Data from both sexes revealed significant improvements in hanging impulse from Z-IP compared to Z-PBS mice at 4-, 6-, and 7-wks post-injection (Fig. 4a, *p ≤ 0.05). Data from male Z-IP (MZ-IP) and Z-PBS (MZ-PBS) mice alone revealed an improvement in MZ-IP at only 8-wks post-injection (Fig. 4b, *p ≤ 0.05). However, female Z-IP (FZ-IP) mice showed a reduction in muscle fatigability compared to female Z-PBS (FZ-PBS) mice at six time points between 5-d and 7-wks post-transplantation (Fig. 4c, *p ≤ 0.05, **p ≤ 0.01). These results suggest that systemic transplantation of young MDSPCs preserves muscle function in progeroid ZMPSTE24-deficient mice, predominantly in female mice.

### Systemic transplantation of young MDSPCs preserves muscle composition and innervation in a sex-specific manner

To determine whether systemic transplantation of young MDSPCs had a therapeutic effect on muscle histopathology, we analyzed cryosections of muscle tissues from Z-IP and Z-PBS mice. The GS isolated from 6-month-old Z-IP and Z-PBS mice were labeled for dystrophin (green, Fig. 4d). The cross-sectional areas of dystrophin + muscle fibers from both sexes combined (Fig. 4e) or MZ-IP and MZ-PBS were not significantly different (Fig. 4f). However, cross-sectional areas of muscle fibers from FZ-IP mice (Fig. 4g; 410.1 µm^2^) were significantly greater than FZ-PBS mice (307.5 µm^2^; *p ≤ 0.05). Frequency distribution analysis of GS muscle fiber cross-sectional areas clearly illustrates fiber size differences between FZ-IP and FZ-PBS mice (Fig. 4h; *p ≤ 0.05, **p ≤ 0.01). Approximately 50% of the dystrophin-positive myofibers in FZ-IP mice had an area > 500 µm^2^ compared to FZ-PBS mice which had > 60% of their myofibers smaller than 400 µm^2^.

**Fig. 5:**
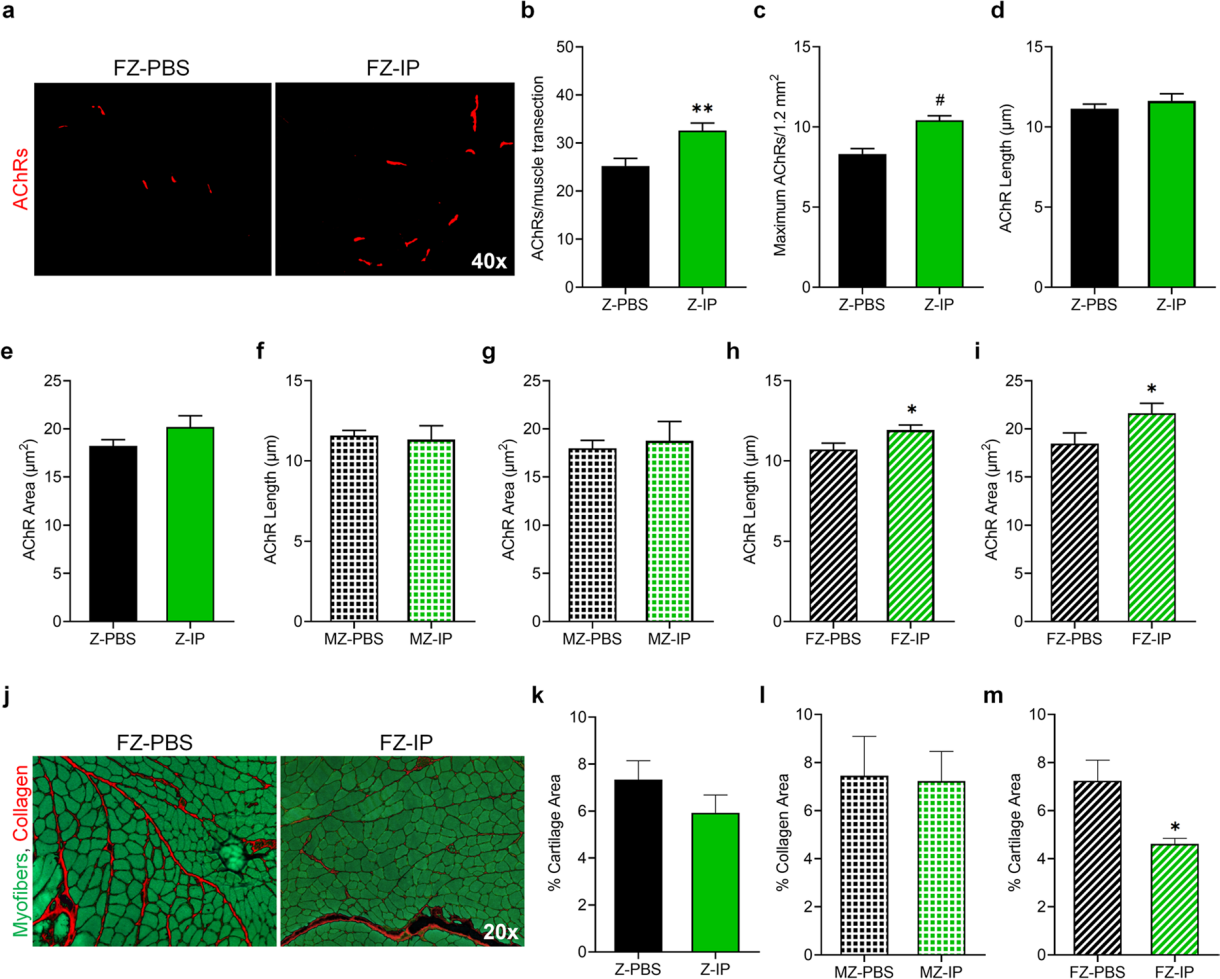
Systemic transplantation of young MDSPCs increases muscle innervation in ZMPSTE24-deficient mice and increases AChR morphometrics and reduces muscle fibrosis in female ZMPSTE24-deficient mice. **a** Representative images of α-bungarotoxin (α-BTX) labeled acetylcholine receptors (AChRs, red) in gastrocnemius muscles from ZMPSTE24-deficient mice intraperitoneally transplanted with either PBS (Z-PBS) or young MDSPCs (Z-IP). **b** Graphs indicating the number of total AChRs per muscle cross section and **c** the average density of AChRs from Z-PBS and Z-IP gastrocnemius muscles. **d** Quantification of AChR length and **e** AChR area from Z-PBS and Z-IP gastrocnemius muscles. **f** Graphs indicating the length and **g** area of AChRs from male Z-PBS (MZ-PBS) and Z-IP (MZ-IP) gastrocnemius muscles. **h** Graphs indicating the length and **i** area of AChRs from female Z-PBS (FZ-PBS) and Z-IP (FZ-IP) gastrocnemius muscles. **j** Representative images of sirius red (red) and fast green (green) stained gastrocnemius muscles from Z-PBS and Z-IP mice. **k** Quantification of percent collagen area in Z-PBS and Z-IP, **l** MZ-PBS and MZ-IP, **m** and FZ-IP and FZ-PBS gastrocnemius muscles. Error bars indicate ± SEM; *n* = 8 to 11 for Z-PBS; *n* = 8 for Z-IP; *n* = 4 to 5 for MZ-PBS, MZ-IP, FZ-PBS, and FZ-IP. *p ≤ 0.05 and **p ≤ 0.01 using one-tailed, unpaired Student’s *t*-test or Welch’s unequal variance *t*-test

To determine the alterations to motoneuron innervation potential from available AChRs within the GS muscle, we examined the quantity and morphology of postsynaptic end plates. AChRs were labeled with α-BTX (red, Fig. 5a, Fig. S2) and revealed a significant increase in the number of AChRs in GS muscles of Z-IP mice compared to Z-PBS mice, whether quantified as per muscle transection (Fig. 5b; 32.6 and 25.2, respectively; **p ≤ 0.01) or density (Fig. 5c; 10.4 and 8.3, respectively; ^#^*p* ≤ 0.001). However, morphometric parameters of AChRs, including length (Fig. 5d) and area (Fig. 5e), were not significantly different between Z-IP and Z-PBS mice or MZ-IP and MZ-PBS (Fig. 5f and g, respectively). However, AChRs in FZ-IP muscles showed a significant increase in length (Fig. 5h; 11.9 µm; *p ≤ 0.05) and area (Fig. 5i; 21.6 µm^2^; *p ≤ 0.05) compared to FZ-PBS muscle AChRs (10.7 µm and 18.5 µm^2^, respectively). These data further suggest that there are sex-related differences in motor unit preservation following systemic transplantation of young MDSPCs.

To assess muscle fibrosis, collagen content was measured in Z-IP and Z-PBS GS muscles by staining cryosections with fast green and sirius red (red; Fig. 5j). While average collagen area was slightly reduced in Z-IP mice (Fig. 5k; 5.9%) compared to Z-PBS (7.3%), there was no significant difference between both sexes combined or MZ-IP and MZ-PBS (Fig. 5l; 7.2% and 7.5%, respectively). However, when separated from male mice, FZ-IP muscle contained significantly less collagen than their FZ-PBS littermates (Fig. 5m; 4.6% and 7.2%, respectively; *p ≤ 0.05). The reduced area of fibrosis demonstrates the capacity for systemic transplantation of young MDSPCs to support proper muscle composition in female mice during aging.

### Systemic transplantation of young MDSPCs preserves mitochondrial respirometry function and fatigue-resistant fiber types in skeletal muscle

**Fig. 6:**
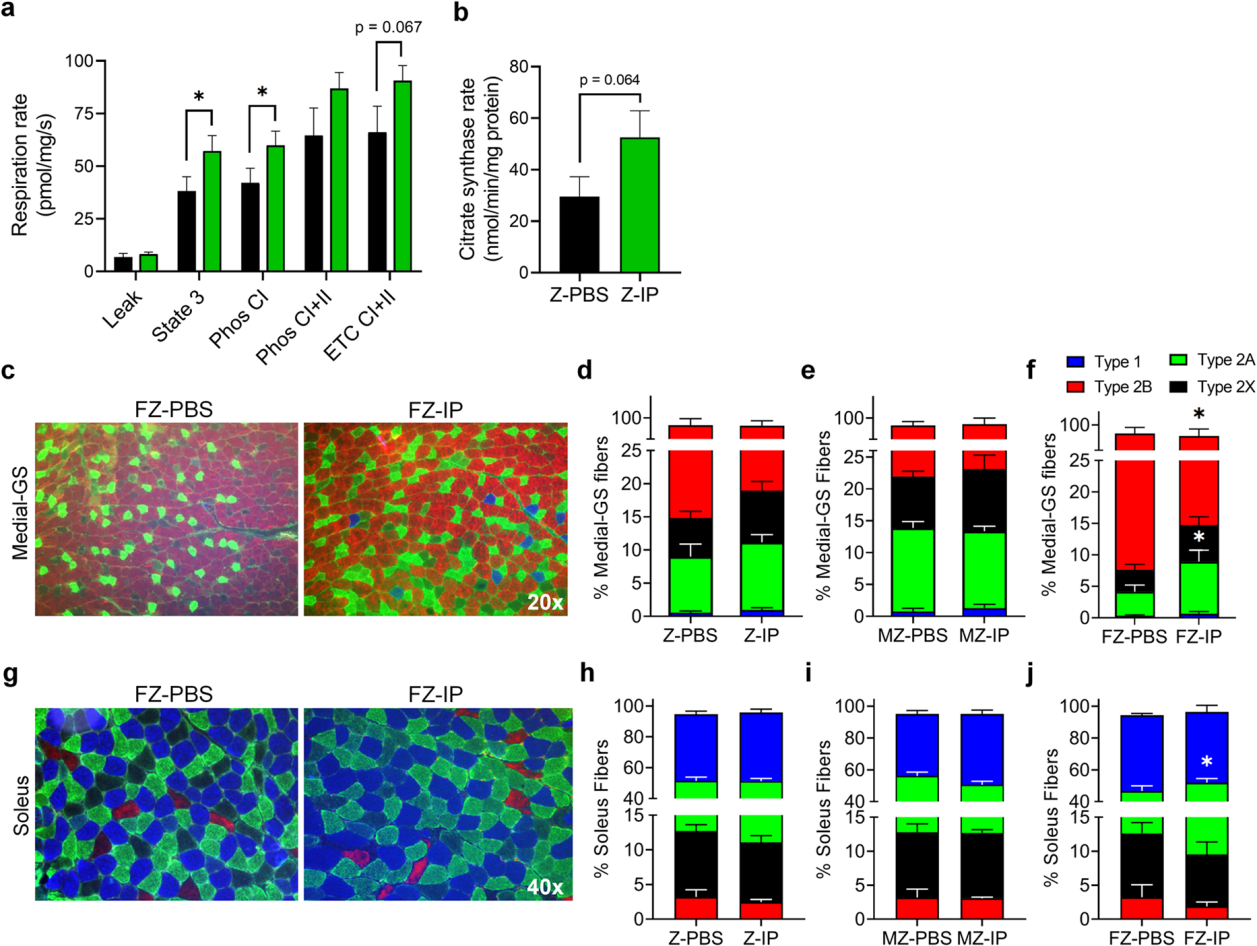
Systemic transplantation of young MDSPCs preserves mitochondrial function in ZMPSTE24-deficient mice and maintains fatigue-resistant fiber types in skeletal muscle of female ZMPSTE24-deficient mice. **a** Analysis of respiration rate and **b** citrate synthase rate measured from quadriceps muscles of ZMPSTE24-deficient mice intraperitoneally injected with either PBS (Z-PBS) or young MDSPCs (Z-IP). **c** Representative images of fiber type labeling in medial gastrocnemius (medial-GS) muscles from female Z-IP and Z-PBS mice. **d** Quantification of medial-GS muscles from Z-PBS and Z-IP mice; **e** male Z-PBS (MZ-PBS) and Z-IP (MZ-IP) mice; and **f** female Z-PBS (FZ-PBS) and Z-IP (FZ-IP) mice immunohistochemically labeled for type 1 (slow fatigue-resistant; blue), type 2A (fast fatigue-resistant; green), type 2X (fast fatigable; black), or type 2B (fast highly fatigable; red). **g** Representative images of fiber type labeling in soleus muscles from Z-PBS and Z-IP mice. **h** Quantification of immunohistochemical fiber type analysis from Z-PBS and Z-IP, **i** MZ-PBS and MZ-IP, and **j** FZ-PBS and FZ-IP soleus muscles. Error bars indicate ± SEM; *n* = 5 (**a** and **b**) and *n* = 8 (**d** and **h**) for Z-PBS; *n* = 7 to 8 for Z-IP; *n* = 4 for MZ-PBS, MZ-IP, FZ-PBS, and FZ-IP. *p ≤ 0.05 using one-tailed, unpaired Student’s *t*-test or Welch’s unequal variance *t*-test

Mitochondrial respirometry states and citrate synthase rates were measured in quadriceps muscles of Z-IP and Z-PBS mice. Comparisons between mitochondrial respiration of Z-IP and Z-PBS muscles demonstrated significantly greater respirometry rates of Z-IP muscle at state 3 and Phos CI states compared to Z-PBS (Fig. 6a; *p ≤ 0.05). There was a strong trend toward increase at the ETC CI + CII state, indicative of maximal electron transport chain capacity; but this was not statistically significant (*p* = 0.067). In the muscles of Z-IP and Z-PBS mice, citrate synthase rates (Fig. 6b; 52.6 and 29.5 nmol/min/mg protein, respectively) were not statistically different, but Z-IP showed a strong trend toward an increase (*p* = 0.064). The ratio of muscle fiber types following systemic transplantation was quantified in the medial-GS (Fig. 6c). There was no change in the ratio of myofiber types between Z-IP and Z-PBS (Fig. 6d) or MZ-IP and MZ-PBS (Fig. 6e) mice. However, FZ-IP muscles exhibited a significantly greater ratio of fatigue-resistant type 2A fibers (8.3%) and significantly fewer fatigable type 2B fibers (80.8%) compared to FZ-PBS muscles (Fig. 6f; 3.8% and 88.9%, respectively; *p ≤ 0.05). Similarly, in the soleus muscle (Fig. 6g), there was no statistical difference in muscle fiber types between Z-IP and Z-PBS (Fig. 6h) or MZ-IP and MZ-PBS (Fig. 6i) mice. Analysis of female soleus muscles demonstrated a significant increase in fatigue-resistant type 2A fibers in FZ-IP mice (42.4%) compared to FZ-PBS littermates (Fig. 6j; 34.1%; *p ≤ 0.05). Taken together, these results indicate systemic transplantation of young MDSPCs into progeroid ZMPSTE24-deficient mice preserves skeletal muscle mitochondrial function and fatigue-resistant fibers in a sex-specific manner, favoring female mice.

## Discussion

Our results illustrate the significant muscular dysfunction in progeroid ZMPSTE24-deficient mice throughout their life span and the sex-specific preservation of muscle structure and function in female mice following systemic transplantation with young MDSPCs. At 6 wks of age, progeroid ZMPSTE24-deficient mice demonstrate a drastic functional deficit in muscle fatigability demonstrated by the four-limb hang test. This test presents a functional measurement with high clinical relevance to mobility and fall prevention in older adults [52–54], has been shown to be more sensitive than other muscle fatigue methods, and ensures scientific rigor [55] for detecting muscle impairments. The functional gap between progeroid and WT littermate controls continued to widen as the mice aged, demonstrating the rapid onset of age-related degeneration that occurs in ZMPSTE24-deficient mice [43, 47, 49, 50, 56]. As observed in other aging models, the skeletal muscle of ZMPSTE24-deficient mice undergoes muscle degeneration with characteristics similar to human sarcopenia [50, 57–59]. These skeletal muscle changes include decreased muscle fiber areas, reduced number and morphology of neuromuscular junction forming AChRs, and inferior mitochondria content and performance. ZMPSTE24-deficient mice also experience a muscle fiber type shift, with the soleus muscle losing fatigue-resistant type 2A fibers and fatigue-resistant fibers overall (type 1 and type 2A combined). Therefore, with a life expectancy of approximately 6 months, ZMPSTE24-deficient mice are a well-suited scientific tool for investigating interventions designed to impact age-related muscle function and structure over the entire life span.

Following systemic transplantation of MDSPCs, reduction of muscle fatigability was not significant when the results from both male and female mice were combined. However, further analysis revealed the extended duration of the functional improvement in cell-transplanted female progeroid mice, in contrast to the singular time point for male mice, which initiated the study’s analysis for sex-specific differences. The reduced muscle fatigability in female MDSPC-transplanted mice is most likely due to the significant increase observed in muscle fiber cross-sectional area, AChR morphology, and fatigue-resistant muscle fiber types, as well as the significant decrease in muscle fibrosis. These molecular and tissue-level improvements were only evident in the female mice, correlating with the functional data.

The quantity of AChRs was significantly greater in MDSPC-transplanted mice compared to control PBS-injected mice even with both sexes combined. However, only the female cell-transplanted mice exhibited significantly greater AChR length and area compared to female control mice. These results may indicate the importance of AChR quality over quantity for motor unit innervation and activation during the hang test, where female mice demonstrated superior improvements. These data add a possible sex-related aspect to the previously shown phenomenon that post-synaptic AChR aggregates display fragmentation and reduced quality in aged animals [2]. We hypothesize that this fragmentation process may be reduced in female mice or the mechanism through which MDSPCs preserve neuromuscular phenotypes might be more effective in female mice. Furthermore, only female mice systemically transplanted with MDSPCs displayed significantly greater muscle fiber areas. The lack of differences in muscle fiber area or AChR morphology evident in male mice or both sexes combined supports our conclusion that systemic transplantation of MDSPCs promotes muscle rejuvenation in a sex-specific manner.

As mammals age, muscle cells deposit greater amounts of collagen, which increases muscle stiffness and reduces muscle performance [17, 60]. Therefore, preventing fibrosis is vital for maintaining strength and mobility in older age. Comparisons of muscles from MDSPC-treated male mice and PBS control mice displayed no differences in collagen content. However, female cell-treated mice showed significantly less fibrosis compared to their age-matched negative control female littermates. A shift toward pro-fibrotic cell phenotypes is often related in aging to senescence-associated secretory phenotypes (SASPs) [61]. Our previous findings demonstrated that systemic transplantation of young MDSPCs reduces SASP markers in articular cartilage [43, 44]. It is possible that similar mechanisms are supporting the reduction of fibrosis in skeletal muscle of female progeroid mice, warranting future investigation.

A large body of work has identified the considerable changes that occur in muscle metabolism throughout the aging process [3, 14, 62–64]. Our results show that mitochondrial respiration rates across all states of the electron transport chain as well as citrate synthase rate, a marker of mitochondrial content [65], are consistently lower in ZMPSTE24-deficient PBS control mice compared to WT mice, demonstrating similar age-related decline in mitochondrial function. Muscles from ZMPSTE24-deficient mice systemically treated with MDSPCs maintained a significantly elevated rate of respirometry through state 3 and Phos CI and displayed a strong trend at the ETC CI + CII state, indicating an increase to the maximal electron transport chain capacity. These results, along with the trending increase of the citrate synthase rate, suggest that MDSPC treatment was able to preserve portions of the muscle metabolism as the mice aged. In addition, a comprehensive assay of muscle fiber types was utilized to identify types 1, 2A, 2B, and 2X [66]. Hybrid fibers were also identified using this method but accounted for a statistically insignificant quantity. We observed a significantly larger ratio of fatigue-resistant type 2A fibers in both gastrocnemius and soleus muscles from female mice transplanted with MDSPCs. The increase specifically of fatigue-resistant muscle fibers is of particular interest since the observed functional improvement was reduced muscle fatigability. A shift toward fatigue-resistant fibers is a very plausible supportive mechanism for the observed functional augmentation. Interestingly, the increase in fatigue-resistant muscle fibers may also be mechanistically related to the preservation of neuromuscular junctions following MDSPCs systemic transplantation, since the degradation rate of neuromuscular junctions is fiber type dependent [67].

Striking differences in muscle function and histology between sexes observed in our study may indicate young MDSPCs secrete or interact with sex-dependent hormones, leading to differences in muscular preservation. Sarcopenia and other muscle wasting diseases have been shown to affect males and females differently, due to higher testosterone levels in men—which is a potent muscle growth stimulator—and higher estrogen levels in women—which tends to support muscle preservation by reducing catabolic activity [68]. For postmenopausal females in particular—in humans and mice—estrogen levels positively correlate with muscle strength in a variety of muscle groups [69]. Hormone levels were not measured in this study but will be quantified in future experiments to consider and correlate this important confounding variable. However, these hormone levels also begin to fluctuate in older age, with a reported variation of approximately 7 months in the onset of cessation of estrous cycles in C57Bl6 mice [70, 71], further complicating identification of sex-dependent mechanisms.

Notably, the young MDSPCs used for transplantation in this study were isolated from a female mouse. Differences in muscle regenerative capacity related to host and donor sex are not a new observation [72]. In fact, previous studies in young mice using local transplantation of MDSPCs showed that skeletal muscle demonstrated the highest regeneration index when donor cells are sex-matched with the host [72]. The lower regeneration index in sex-mismatched mice was notably not due to immune rejection, since differences were also identified when using severe combined immunodeficient (SCID) host mice [72]. Therefore, it is possible that the sex-specific results of female mouse improvement in this study are, at least in part, due to the sex-match between host and donor. Future experiments analyzing the levels of circulating factors before and after treatments may provide further insight into whether correlative changes are found between function and sex-specific hormones, more traditional regenerative growth factors, or both. Similarly, comparative analysis of the secretome from MDSPCs isolated from female mice to those isolated from male mice will provide further guidance on whether female cells ultimately secrete higher levels of regenerative factors or if sex-specific hormones mediate the primary differences.

It has also been reported that the hormonal milieu of female host skeletal muscle is better suited for donor cell transplantation than male host skeletal muscle [72, 73]. While both male and female mice undergo muscle and functional loss with age, the sex-specific mechanisms vary between sexes [68]. In skeletal muscle, testosterone is a potent anabolic signaling factor, and estrogen is a strong inhibitor of catabolic activity; therefore, the steady decline of these hormones leads down two distinct pathways for muscle wasting [68]. It is possible that the catabolic mechanisms involved in female-specific muscle wasting are more readily reversible, allowing for the shift toward a healthier regenerative environment. This supports our hypotheses that the observed sex differences occur because MDSPCs isolated from females are superior donors for muscle regeneration and that female skeletal muscle maintains a better microenvironment for regeneration. Indeed, our findings highlight for the first time the sex-specific differences in neuromuscular preservation following a systemic cell therapy in an animal model of aging.

Limitations to this study include the lack of a more direct motor function testing, such as with a treadmill or DigiGait, due to the difficulty in measuring functional outcomes in the progeroid ZMPSTE24-deficient mice. The pronounced size reduction of these progeroid mice progressively limits their ambulation capacity starting from a relatively young age, making longitudinal gait or treadmill testing impractical. In addition, due to breeding issues and cohort enrollment restrictions, requiring at least two sex-matched homozygous ZMPSTE24-deficient mice from a single litter, the sex-specific groups are relatively small. Though all mice in both cohorts having a littermate counterpart in the juxtaposing group certainly makes comparisons stronger by limiting additional environmental confounding factors. Similarly, sex-specific comparisons with mitochondrial respirometry were not possible due to disqualification of some PBS samples following the cytochrome c test, which determined the mitochondrial outer membranes were already over permeabilized and no longer intact. This reduction to female PBS samples left sex-specific comparisons underpowered and were therefore not included.

Collectively, our results revealed for the first time that MDSPCs exhibit sex-specific differences in neuromuscular rejuvenation following systemic transplantation, and these differences can occur while preserving neuromuscular function throughout the life span of aged mice. This evidence emphasizes the continued need to validate regenerative mechanisms in both male and female systems and may have significant implications for how we approach healthy neuromuscular aging moving forward.

### Supplementary Information

Below is the link to the electronic supplementary material.

Supplementary file1 (DOCX 764 KB)
