# Supplementary material for: Sex-specific preservation of neuromuscular function and metabolism following systemic transplantation of multipotent adult stem cells in a murine model of progeria
Source: GeroScience. 2023 Aug 3;46(1):1285–302. doi: 10.1007/s11357-023-00892-5 (PMC10828301; doi:10.1007/s11357-023-00892-5)
Supplement: Supplementary file 1 — Supplementary file1 (DOCX 764 KB) [file 11357_2023_892_MOESM1_ESM.docx]

**Supplementary Information**

**GeroScience**

**Sex-specific preservation of neuromuscular function and metabolism following systemic transplantation of multipotent adult stem cells in a murine model of progeria**

Seth D. Thompson^1,2,3,^*, Kelsey L. Barrett^1^, Chelsea L. Rugel^1,2,3^, Robin Redmond^1^, Alexia Rudofski^1^, Jacob Kurian^4^, Jodi L. Curtin^1^, Sudarshan Dayanidhi^1,2^, Mitra Lavasani^1,2,3,^*

^1^Shirley Ryan AbilityLab, Chicago, IL, 60611, USA

^2^Department of Physical Medicine and Rehabilitation, Northwestern University, Chicago, IL, 60611, USA

^3^Northwestern University Interdepartmental Neuroscience (NUIN) Graduate Program, Northwestern University, Chicago, IL, 60611, USA

^4^Department of Biomedical Engineering, Northwestern University, Chicago, IL, 60611, USA

***Correspondence should be addressed to:** Dr. Mitra Lavasani ([mlavasani@sralab.org](mailto:mlavasani@sralab.org)) or Dr. Seth Thompson ([seththompson2018@u.northwestern.edu](mailto:seththompson2018@u.northwestern.edu)), Shirley Ryan Abilitylab, 355 E. Erie St, Chicago, IL 60611, USA.

**Supplemental Figures**

**
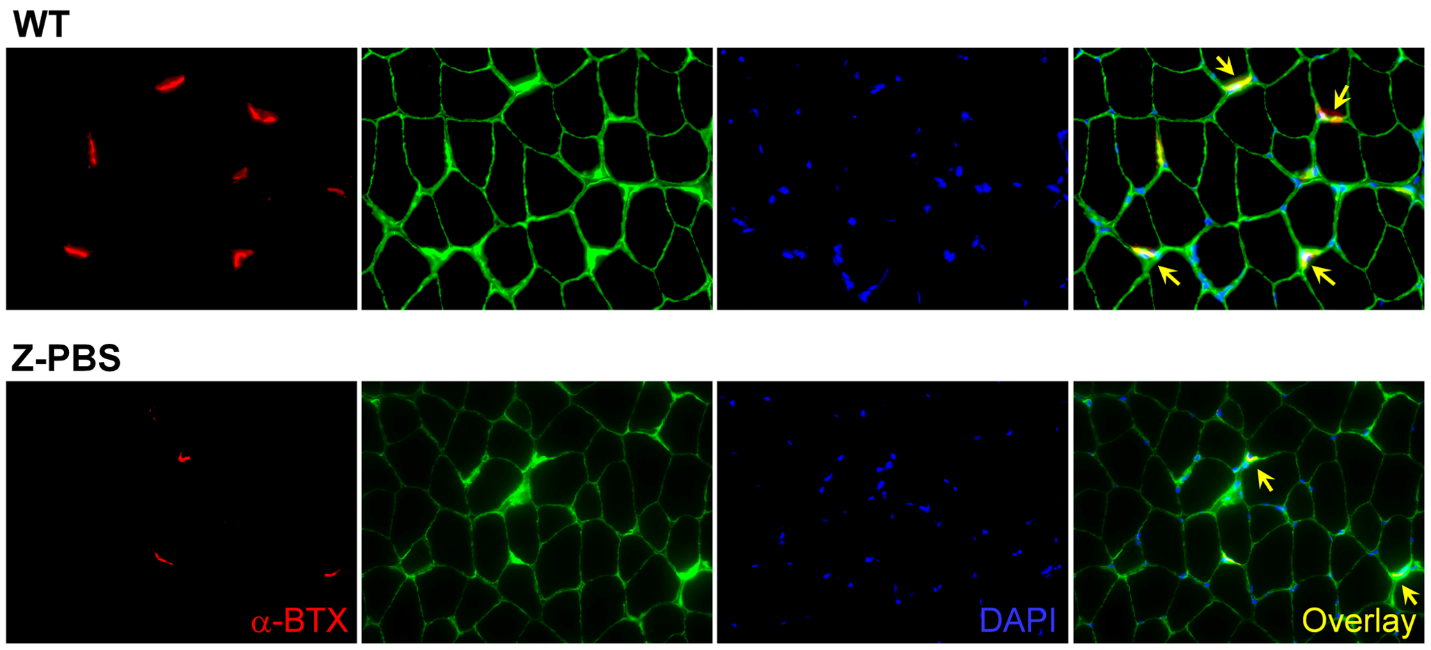
**

**Fig S1: Progeroid ZMPSTE24-deficient mice exhibit reduced innervation.** Representative images of gastrocnemius muscles from wildtype (WT) and ZMPSTE24-deficient mice intraperitoneally transplanted with PBS (Z-PBS) labeled with α-bungarotoxin (α-BTX, red) for AChRs, antibodies against dystrophin (Dys, green) for muscle fibers, and DAPI (blue) for nuclei. Yellow arrows indicate the AChRs overlapping with dystrophin+ myofibers, in the merged images. Magnification is 40x.

**
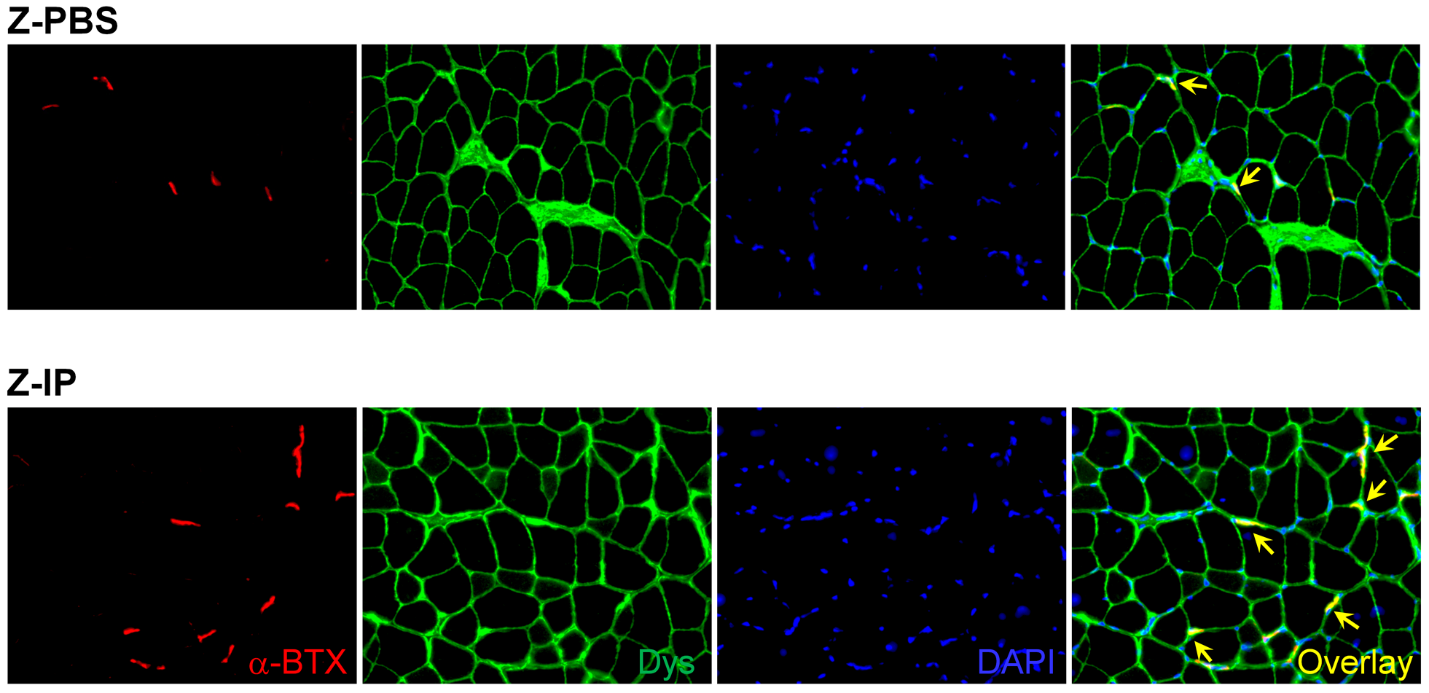
**

**Fig S2: Systemic transplantation of young MDSPCs preserves muscle innervation in progeroid ZMPSTE24-deficient mice.** Representative images of gastrocnemius muscles, from ZMPSTE24-deficient mice intraperitoneally transplanted with either PBS (Z-PBS) or young MDSPCs (Z-IP), labeled with α-bungarotoxin (α-BTX, red) for AChRs, antibodies against dystrophin (Dys, green) for muscle fibers, and DAPI (blue) for nuclei. Yellow arrows indicate the AChRs overlapping with dystrophin+ myofibers, in the merged images. Magnification is 40x.

**Table S1.** Experiment Animal Numbers

| **Figure** | **Cohort** | **Total (n)** | **Male (n)** | **Female (n)** |
| --- | --- | --- | --- | --- |
| 2a | WT | 8 | 4 | 4 |
| 2a | Z-PBS | 8 | 4 | 4 |
| 2c | WT | 7 | 4 | 3 |
| 2c | Z-PBS | 8 | 4 | 4 |
| 2d | WT | 7 | 4 | 3 |
| 2d | Z-PBS | 8 | 4 | 4 |
| 2f | WT | 8 | 4 | 4 |
| 2f | Z-PBS | 11 | 4 | 7 |
| 2g | WT | 8 | 4 | 4 |
| 2g | Z-PBS | 10 | 4 | 6 |
| 2h | WT | 8 | 4 | 4 |
| 2h | Z-PBS | 8 | 4 | 4 |
| 2i | WT | 8 | 4 | 4 |
| 2i | Z-PBS | 8 | 4 | 4 |
|  |  |  |  |  |
| 3a | WT | 8 | 4 | 4 |
| 3a | Z-PBS | 5 | 3 | 2 |
| 3b | WT | 8 | 4 | 4 |
| 3b | Z-PBS | 5 | 3 | 2 |
| 3d | WT | 8 | 4 | 4 |
| 3d | Z-PBS | 8 | 4 | 4 |
| 3f | WT | 8 | 4 | 4 |
| 3f | Z-PBS | 8 | 4 | 4 |
|  |  |  |  |  |
| 4a | Z-PBS | 8 | 4 | 4 |
| 4a | Z-IP | 7 | 4 | 3 |
| 4b | MZ-PBS | 4 | 4 | 4 |
| 4b | MZ-IP | 4 | 4 | 4 |
| 4c | FZ-PBS | 4 | 0 | 4 |
| 4c | FZ-IP | 3 | 0 | 3 |
| 4e | Z-PBS | 8 | 4 | 4 |
| 4e | Z-IP | 8 | 4 | 4 |
| 4f | MZ-PBS | 4 | 4 | 0 |
| 4f | MZ-IP | 4 | 4 | 0 |
| 4g | FZ-PBS | 4 | 0 | 4 |
| 4g | FZ-IP | 4 | 0 | 4 |
| 4h | FZ-PBS | 4 | 0 | 4 |
| 4h | FZ-IP | 4 | 0 | 4 |
|  |  |  |  |  |
|  |  |  |  |  |
| 5b | Z-PBS | 11 | 4 | 7 |
| 5b | Z-IP | 8 | 4 | 4 |
| 5c | Z-PBS | 10 | 4 | 6 |
| 5c | Z-IP | 8 | 4 | 4 |
| 5d | Z-PBS | 8 | 4 | 4 |
| 5d | Z-IP | 8 | 4 | 4 |
| 5e | Z-PBS | 8 | 4 | 4 |
| 5e | Z-IP | 8 | 4 | 4 |
| 5f | MZ-PBS | 4 | 4 | 0 |
| 5f | MZ-IP | 4 | 4 | 0 |
| 5g | MZ-PBS | 4 | 4 | 0 |
| 5g | MZ-IP | 4 | 4 | 0 |
| 5h | FZ-PBS | 4 | 0 | 4 |
| 5h | FZ-IP | 4 | 0 | 4 |
| 5i | FZ-PBS | 4 | 0 | 4 |
| 5i | FZ-IP | 4 | 0 | 4 |
| 5k | Z-PBS | 9 | 4 | 5 |
| 5k | Z-IP | 8 | 4 | 4 |
| 5l | MZ-PBS | 4 | 4 | 0 |
| 5l | MZ-IP | 4 | 4 | 0 |
| 5m | FZ-PBS | 5 | 0 | 5 |
| 5m | FZ-IP | 4 | 0 | 4 |
|  |  |  |  |  |
| 6a | Z-PBS | 5 | 3 | 2 |
| 6a | Z-IP | 7 | 4 | 3 |
| 6b | Z-PBS | 5 | 3 | 2 |
| 6b | Z-IP | 7 | 4 | 3 |
| 6d | Z-PBS | 8 | 4 | 4 |
| 6d | Z-IP | 8 | 4 | 4 |
| 6e | MZ-PBS | 4 | 4 | 0 |
| 6e | MZ-IP | 4 | 4 | 0 |
| 6f | FZ-PBS | 4 | 0 | 4 |
| 6f | FZ-IP | 4 | 0 | 4 |
| 6h | Z-PBS | 8 | 4 | 4 |
| 6h | Z-IP | 8 | 4 | 4 |
| 6i | MZ-PBS | 4 | 4 | 0 |
| 6i | MZ-IP | 4 | 4 | 0 |
| 6j | FZ-PBS | 4 | 0 | 4 |
| 6j | FZ-IP | 4 | 0 | 4 |

Provided are the numbers of individual mice used, per cohort, in each figure that presents graphical data. Total mouse numbers are further divided into number of male and female mice to demonstrate balance in experiments including both sexes. WT = wildtype. Z-PBS = ZMPSTE24-deficient mice transplanted with PBS. Z-IP = ZMPSTE24-deficient mice transplanted with young MDSPCs. MZ-PBS = Male ZMPSTE24-deficient mice transplanted with PBS. MZ-IP = Male ZMPSTE24-deficient mice transplanted with young MDSPCs. FZ-PBS = Female ZMPSTE24-deficient mice transplanted with PBS. FZ-IP = Female ZMPSTE24-deficient mice transplanted with young MDSPCs.
